# Observations on Some Topical Remedies

**Published:** 1811-06

**Authors:** John Ring

**Affiliations:** New Street, Hanover Square


### *To the Editors of the Medical and Physical Journal.*

Gentlemen,

In my letter, inserted in a former number of your Journal, containing a valuable and interesting communication from Mr. Park, on the state of vaccination at Liverpool, my signature was omitted by mistake. This would not be material, did I not consider myself responsible for the accuracy of the statement, in the concluding part of that letter.

I now request the favour of you to insert a few miscellaneous observations on some topical remedies, which I have found beneficial; and which deserve to be more generally known.

### *Styptics.*

The most powerful styptic that I have ever used, is powder of charcoal. Having seen the remains of a small box of a black powder brought from Flanders, which was sold at a high price, and reputed to be possessed of extraordinary properties as a styptic, I suspected it to be charcoal; and had some charcoal powdered, which, on trial, proved equal, if not superior, to the original. In bleedings of the nose, it may be applied by means of tents, first moistened with water, and then dipped in this powder; but in slight cases it has answered, by being taken like snuff. In other cases it is to be sprinkled on the part affected; and retained there by means of a compress.

### *On Ophthalmia and Psorophthalmia.*

The advantage of metallic oxyds, particularly that of mercury, in obstinate cases of this kind, is well ascertained. The ung. hydrarg. nitratis is a good preparation in skilful hands, when cautiously cautiously applied, either with the finger or a camel hair pencil; but it *is* too strong for popular use. The following, which is much milder, has proved so efficacious, and given such general satisfaction, that I deem it an indispensable duty to make it public. R. Hydrarg. nitrico-oxydi. gr. xv. butyri. ℥j. M.

The edges of the eyelids should be anointed with this ointment every night; or every night and morning.

### *On the Favus.*

I several years ago published in this Journal, some remarks on the treatment of tinea, the common effect of favus when it takes place on the scalp; I now beg leave to add, that when this eruption occurs in the face, where it is the common effect of a cold, nothing has appeared to be more efficacious than the ung. picis liquidæ; but a certain degree of caution is necessary in its use; otherwise it will prove too stimulating.

### *On Excoriations behind the Ears.*

The following is the best remedy that I have ever found for this complaint. R. Plumbi superacet. ℈j. adipis præpar. ℥j. M. This is to be spread on lint. The disorder often extends over the ears; but is easily cured by this application. The sores must not be washed.

### *On Herpetic eruptions.*

The best remedies for these eruptions are ointments prepared from hydrarg. præcip. alb. with a small quantity of hydrarg. oxymur. and lard.

The ung. zinci has also been found serviceable in such eruptions; particularly in those of the hands, or other irritable parts.

A lotion similar to the nostrum, sold under the name of Gowlard's lotion, has cured some herpetic eruptions, which were so troublesome as to render life intolerable. It is as follows:—R. Amygd. amar. decort. ʒiij. tere in mortario marmoreo cum aquæ. ℥viij. et adde hydrarg. oxymur. gr. viij. in spiritûs rosmarini. ʒij. priùs solutæ. Fiat lotio.

### *Oleum Ricini.*

This important article of the Materia Medica is much improved by filtering; and it is of some consequence to ascertain the best mode of exhibiting it, particularly in delicate stomachs. I have found it most convenient to give it in sugar and water. A little moist sugar should first be dissolved in tepid water, in a tea-cup; and stirred with the spoon in which the oil is to be measured. By this method the oil is prevented from sticking to the the tea-cup, or the spoon; and not only the dose will be more exact; but it will float loosely on the surface of the vehicle, and be more easily taken.

I have almost always found this the best aperient for puerperal patients; many of whom had in vain tried to take the oil in other forms.

I cannot conclude without observing, that this department of physic stands in need of reform. An aperient should always be given within forty-eight hours after delivery, unless the patient has had a stool. It is proper to direct it on the morning of the third day inclusively. A table-spoonful of the castor oil is in general sufficient; and ought not to be repeated in less than twenty-four hours, lest it should operate too much. If occasion requires, its operation should be promoted by a clyster.

I have frequently heard, and heard with great concern, of cases in which women have been suffered to remain without a stool, and even without an attempt to procure one, six or seven days. This gross and shameful neglect can only arise from the practice being still, in some measure, in bad hands. It is, indeed, an Augean stable, and ought to be cleansed. Many of the male sex, who now practise that branch, were never educated to the medical profession.

To these remarks it may be added, that during the month, no woman should be suffered to go more than twenty-four hours without a stool; and that the dose of the oil should be gradually diminished.

I am, Gentlemen,

Yours, &c.

*New Street, Hanover Square,* JOHN RING.

*April* 16, 1811.

